# Ectopic overexpression of a type-II DGAT (CeDGAT2-2) derived from oil-rich tuber of *Cyperus esculentus* enhances accumulation of oil and oleic acid in tobacco leaves

**DOI:** 10.1186/s13068-021-01928-8

**Published:** 2021-03-23

**Authors:** Yu Gao, Yan Sun, Huiling Gao, Ying Chen, Xiaoqing Wang, Jinai Xue, Xiaoyun Jia, Runzhi Li

**Affiliations:** 1 College of Agriculture, Institute of Molecular Agriculture and Bioenergy, Shanxi Agricultural University, Taigu, 030801 Shanxi China; 2 College of Life Sciences, Shanxi Agricultural University, Taigu, 030801 Shanxi China

**Keywords:** Type II diacylglycerol acyltransferase (CeDGAT2-2), Triacylglycerol (TAG) biosynthesis, *Cyperus esculentus*, *Saccharomyces cerevisiae* mutant H1246, Transgenic tobacco (*Nicotiana tabacum* L.), Oil accumulation in non-seed tissues

## Abstract

**Background:**

Engineering triacylglycerol (TAG) accumulation in vegetative tissues of non-food crops has become a promising way to meet our increasing demand for plant oils, especially the renewable production of biofuels. The most important target modified in this regard is diacylglycerol acyltransferase (DGAT) enzyme responsible for the final rate-limiting step in TAG biosynthesis. *Cyperus esculentus* is a unique plant largely accumulating oleic acid-enriched oil in its underground tubers. We speculated that DGAT derived from such oil-rich tubers could function more efficiently than that from oleaginous seeds in enhancing oil storage in vegetative tissues of tobacco, a high-yielding biomass crops.

**Results:**

Three *CeDGAT* genes namely *CeDGAT1*, *CeDGAT2-1* and *CeDGAT2-2* were identified in *C. esculentus* by mining transcriptome of developing tubers. These *CeDGATs* were expressed in tissues tested, with *CeDGAT1* highly in roots, *CeDGAT2-1* abundantly in leaves, and *CeDGAT2-2* predominantly in tubers. Notably, *CeDGAT2-2* expression pattern was in accordance with oil dynamic accumulation during tuber development. Overexpression of *CeDGAT2-2* functionally restored TAG biosynthesis in TAG-deficient yeast mutant H1246. Oleic acid level was significantly increased in *CeDGAT2-2* transgenic yeast compared to the wild-type yeast and *ScDGA1-*expressed control under culture with and without feeding of exogenous fatty acids. Overexpressing *CeDGAT2-2* in tobacco led to dramatic enhancements of leafy oil by 7.15- and 1.7-fold more compared to the wild-type control and plants expressing *Arabidopsis* seed-derived *AtDGAT1*. A substantial change in fatty acid composition was detected in leaves, with increase of oleic acid from 5.1% in the wild type to 31.33% in *CeDGAT2-2-*expressed tobacco and accompanied reduction of saturated fatty acids. Moreover, the elevated accumulation of oleic acid-enriched TAG in transgenic tobacco exhibited no significantly negative impact on other agronomic traits such as photosynthesis, growth rates and seed germination except for small decline of starch content.

**Conclusions:**

The present data indicate that CeDGAT2-2 has a high enzyme activity to catalyze formation of TAG and a strong specificity for oleic acid-containing substrates, providing new insights into understanding oil biosynthesis mechanism in plant vegetative tissues. Overexpression of *CeDGAT2-2* alone can significantly increase oleic acid-enriched oil accumulation in tobacco leaves without negative impact on other agronomy traits, showing *CeDGAT2-2* as the desirable target gene in metabolic engineering to enrich oil and value-added lipids in high-biomass plants for commercial production of biofuel oils.

**Supplementary Information:**

The online version contains supplementary material available at 10.1186/s13068-021-01928-8.

## Background

Since the industrial revolution, modern society has been relying heavily on fossil oil as a source to provide a cheap mean of energy and a wide range of tailored oleochemicals. With the continued growth of world population and environmental concerns associated with excessive consumption of petroleum, there is an increasing need to develop more sustainable, environmentally friendly alternatives to reduce our reliance on dwindling crude oil supplies. Plant-derived oils predominantly enriched in triacylglycerol (TAG), one of the most energy-dense forms of carbon found in nature, are chemically similar to the long-chain hydrocarbons of fossil oil and thus represent outstanding renewable sources of carbon-neutral raw materials [1–3]. In fact, a significant proportion of vegetable oil from common oilseed crops such as soybean and canola is already used as feedstocks for biodiesel production [4]. However, such large-scale non-food application of plant oil increases a risk of world food security because those established oil crops normally serve as the major resource for human food and animal feed. Moreover, government policies and regulations for increased usage of renewable fuels brought to bear additional pressure on agricultural production systems [5, 6]. To overcome this confliction between food and non-food uses of plant oils, increasing interest has been focused on developing novel, dedicated, non-food bioenergy crops by genetic engineering vegetative tissues of high-biomass crops such as tobacco, sorghum, sugarcane, and potato for producing large amounts of energy-dense oils [7].

Tobacco (*Nicotiana tabacum*) plant has advantages to be developed as biofuel plant. First of all, tobacco is an important non-food cash crop which can generate up to 170 tons/ha of green bio-tissue (leaves and stems). When grown for bioenergy, tobacco can produce large amounts of relatively low-cost biomass feedstock [8]. Secondly, it is reported that the oil content in tobacco leaves is 1.7–4% (dry weight, DW) [9]. The leaf oil can be extracted as fatty acid esters used for biofuel production [10]. Thirdly, tobacco is the easiest plant to be genetically engineered, with highly reproductive and facilitating rapid scale-up. It is recognized that tobacco is a promising platform for “energy factory”. Additionally, tobacco can also be used as a model for biofuel production to utilize other high-biomass plants [11]. Therefore, tobacco is one of the desirable targets for seeking alternative methods to produce bioenergy. Growing effects are focusing on the development of non-food energy crops and the sustainable commercialization of bioenergy through increasing oil content in tobacco and other high-biomass plants.

For these approaches in tobacco and other high-biomass plants, a number of genes involved in TAG biosynthesis and its transcription regulation as well as competitive pathways have been employed as targets for gene modification to enrich oil accumulation in plant leaves and other vegetative organs [12–14]. These genes include *acetyl-CoA carboxylase* gene (*ACCase*), *diacylglycerol acyltransferase* (*DGAT*), *oleosin*, *WRINKLED1*(*WRI1*), *LEAFY COTYLEDON1/2* (*LEC1/2*), *ADP-glucose pyrophosphorylase* (*AGPase*) and *peroxisomal ABC transporter1* (*PXA1*). For example, the simultaneous expression of *WRI1*, *DGAT1* and *Oleosin* genes resulted in enhancement of TAG level to 15% of leaf DW in tobacco leaves, accompanied by a 75% reduction of starch content in mature leaves and plant height decrease [15, 16]. Co-expression of *WRI1*, *DGAT1* and *Oleosin* in combination with simultaneous silencing of *AGPase* and *PXA1* increased TAG levels in leaves and stems of the transgenic sugarcane up to 1.9% and 0.9% of DW, respectively, by 95- and 43-fold elevation compared to the untransformed controls [17]. Overexpressing *Arabidopsis WRI1* and *DGAT1* as well as sesame (*Sesamum indicum*) *Oleosin* in potatoes led to TAG accumulation up to 3.3% of tuber DW, increased by over 100-fold compared to the control [18]. Such lipid increase in the transgenic tubers was associated with a large reduction of starch accumulation and an elevation in soluble sugars. Recently, sorghum (*Sorghum bicolor*), a C4 monocot crop, was also used as the target to be engineered as a dedicated biomass oil crop. The combined overexpression of *Umbelopsis ramanniana DGAT2a*, corn *WRI1* and sesame *Oleosin-L* increased TAG accumulation up to levels between 3 and 8.4% of DW in sorghum leaves with lots of oil droplets visible within mesophyll cells [14]. Of those genes used in this field, *DGAT* is always selected as the key gene to be overexpressed in high-biomass tissues to enrich oil accumulation. DGAT enzymes catalyze diacylglycerol (DAG) to form triacylglycerol (TAG) by transferring an acyl group from acyl-CoA to integrate into *sn*-3 position of DAG molecule, acting as the rate-limiting enzymes for TAG biosynthesis [19]. To date, at least, three DGAT families were identified, namely DGAT1, DGAT2 and DGAT3, respectively, showing less sequence homology among them, with each having multiple members [20]. Generally, DGAT1 is a key enzyme for TAG biosynthesis in oil seeds and fruits [21, 22]. Some members of DGAT2 family have substrate specificity for unusual fatty acids (UFAs) such as eleostearic acid [23], ricinoleic acid [24], and epoxidized fatty acids [25]. Although DGAT1 and DGAT2 are localized at different subdomains in endoplasmic reticulum and have non-redundant functions in TAG synthesis in tung tree [26], DGAT1 and DGAT2 are also identified to have overlapping functions in TAG formation in olive plant [27]. Unlike integral membrane-bound DGAT1 and DGAT2, DGAT3 is a soluble protein enzyme participating in the cytosolic pathway of TAG synthesis [28, 29].

A number of reports showed that different DGATs had differential effects on oil accumulation in the heterogeneous expression hosts. For instance, the overexpression of *Arabidopsis DGAT1* resulted in 7-fold increase in TAG levels in tobacco [30]. The constitutive expression of a *DGAT2* derived from a microalga led to a 25-fold elevation of TAG accumulation in vegetative tissues of *Arabidopsis* [13]. A 5-fold increase of oil content in transgenic tobacco leaves was achieved by ectopic overexpression of *VgDGAT1a* isolated from developing seeds of *Vernonia galamensis* [31]. Apparently, to achieve industrially relevant levels of storage oil in vegetative tissues, it is much needed to identify genes encoding DGATs with high enzyme activity and efficiency for oil enrichment in non-seed organs when expressed in heterogeneous hosts.

So far, most of *DGAT* genes used in this regard are isolated from seed rich in oil. No *DGAT* from vegetative tissues accumulated high level of oil was employed in such engineering to enrich oil content in high-biomass plants. Naturally, certain plants can accumulate oils in non-seed tissues to various degrees, such as 30% oil in avocado mesocarp [32], 10% oil in stem phloem tissue of the ‘oil firewood’ *Tetraena mongolica* [33], and ~ 30% oil in underground storage tubers of *Cyperus esculentus*, namely yellow nutsedge or tigernut [34]. Particularly, *C. esculentus* is a specific oil crop with oil-enriched tubers, strikingly different to common underground crops such as potato, sweet potato and cassava which exclusively contain storage carbohydrates. More importantly, the tuber oil contains 60–75% of oleic acid (18:1Δ9), much higher than that in common oilseeds such as soybean (~ 25%), rape (~ 19%), peanut (~ 40%) and corn oil (~ 24%). Such tuber oil containing high level of monounsaturated FA and low content of polyunsaturated FAs has high stability and resistance to oxidation [35], which is healthier edible oil and also the desirable feedstock for biodiesel. However, the mechanisms responsible for such high oil and oleic acid production in *C. esculentus* tubers are not well characterized.

We hypothesize that DGAT members in *C. esculentus* may have higher enzyme activity and substrate specificity for oleic acid, contributing crucially to the high accumulation of oleic acid and total oil in the storage tubers. Such DGAT derived from non-seed tissues rich in oil may function much effectively in boosting oil accumulation and improving FA composition in heterogeneous vegetative plant tissues. Therefore, in this study, genome-wide identification of *DGAT* genes was performed in *C. esculentus* based on our transcriptome data. Three *DGAT* genes namely *CeDGAT1*, *CeDGAT2-1* and *CeDGAT2-2* were cloned from *C. esculentus* tubers, followed by expression profiling in various tissues and different stages of developing tubers. Their function assay was conducted by expressing them in TAG-deficient yeast mutant H1246, respectively. Finally, *CeDGAT2-2* was constitutively expressed in tobacco plants to evaluate its function and efficiency in oil enrichment in vegetative tissues, showing that CeDGAT2-2 had a strong substrate preference for oleic acid and significantly increased oleic acid and TAG levels in tobacco non-seed biomass by  2-fold more compared to the seed-derived AtDGAT1. The transgenic tobacco plants exhibited no negative phenotypes for other agronomy traits. Clearly, CeDGAT2-2 can be used as the desirable target gene in metabolic engineering of dedicated biomass oil crops for sustainable production of biofuel oils.

## Results

### Identification of DGATs from *Cyperus esculentus*

To identify CeDGAT which may function in TAG biosynthesis in tuber of *C. esculentus,* the developing tubers were used to generate transcriptome data in our laboratory. One *DGAT1* (*CeDGAT1*) and two *DGAT2* (*CeDGAT2-1* and *CeDGAT2-2*) gene cDNAs with whole ORFs were obtained by mining this transcriptome data (the cDNA sequences are shown in Additional file 1). The three *CeDGAT* cDNAs were then cloned by PCR from the mixed tissue sample of *C. esculentus. CeDGAT1* encodes a mature protein consisting of 502 amino acid (aa) residues while *CeDGAT2-1* and *CeDGAT2-2* encode the deduced proteins with 338 aa and 317 aa, respectively. Functional domain analysis using Conserved Domain Database (CDD) in NCBI showed that both AtDGAT1 and CeDGAT1 contain a PLN02401 region, a domain typical of membrane-bound O-acyltransferase (MBOAT) superfamily. Both AtDGAT2 and CeDGAT2s have a PLN02783 region, a domain typical of lysophospholipid acyltransferase (LPLAT) superfamily. CeDGAT1 protein containing eight transmembrane helices is predicted to be located in the endoplasmic reticulum (ER). Two CeDGAT2s only have one transmembrane helice, and they are also predicted to be located in cellular ER (Additional file 2).

**Fig. 1:**
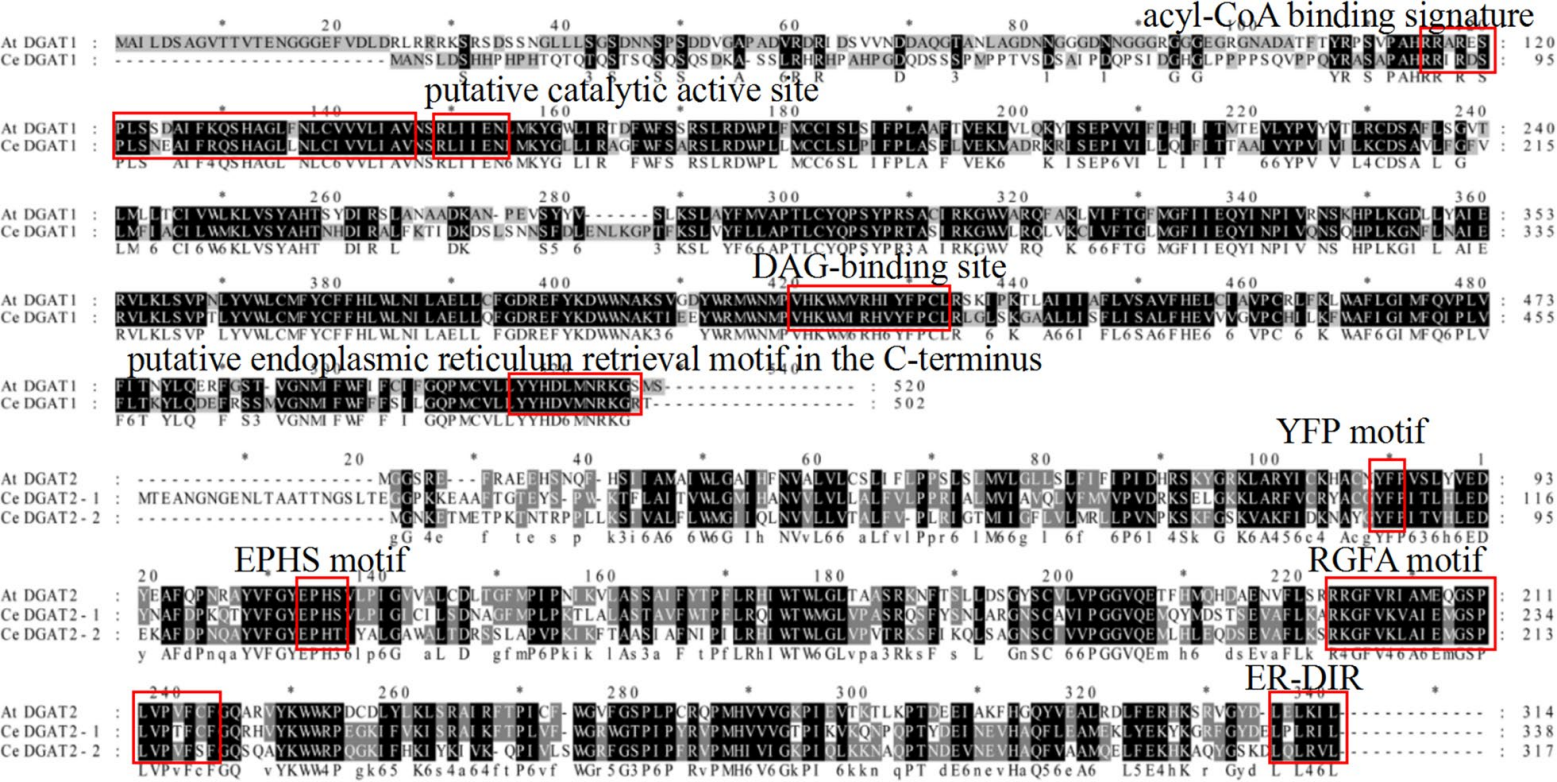
Protein sequence alignment of CeDGAT1 with AtDGAT1(NP_179535), and CeDGAT2s with AtDGAT2(AEE78802). The alignment was performed using the MEGA 7.0 program. Amino acid conservation is marked by different black shades. The conserved motifs are boxed in red

**Fig. 2:**
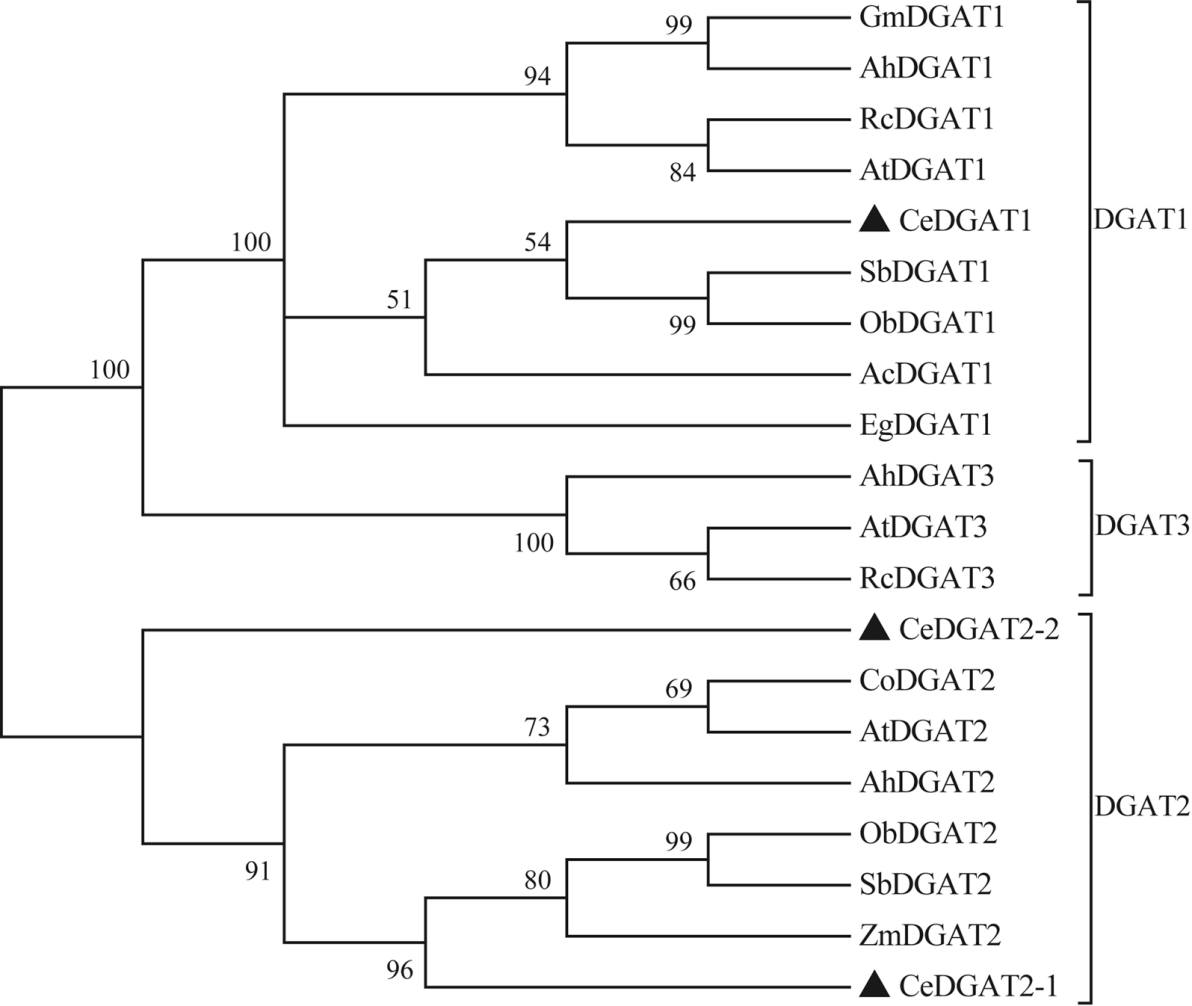
Phylogenetic tree of CeDGATs with other plant DGAT proteins. Phylogenetic tree was generated using MEGA 7.0 by the neighbor-joining method. Percentage values on each branch represent the corresponding bootstrap probability. Protein sequences used for phylogenic analysis included: AcDGAT1 (XP_020097889.1), ObDGAT1 (XP_006657059.1), SbDGAT1 (XP_021304830.1), EgDGAT1 (XP_029119023.1), RcDGAT1 (ACB30543), AhDGAT1 (AGT57761.1), GmDGAT1 (BAE93461.1), AhDGAT2 (AEO11788.1), SbDGAT2 (XP_002452652.1), ObDGAT2 (XP_015689275.1), ZmDGAT2 (AQL03438.1), CoDGAT2 (ATQ37962.1), AhDGAT3 (AAX62735.1), RcDGAT3 (EEF43203.1), AtDGAT3 (Q9C5W0.2). The three CeDGAT proteins were highlighted by the black arrowheads

The identity of CeDGAT1 and AtDGAT1 was 61% while CeDGAT2-1 and CeDGAT2-2 share 52% and 40% of identity with AtDGAT2, respectively. BLAST analysis showed that CeDGAT1 and CeDGAT2 proteins have less than 22% similarity to each other in amino acid sequence. The protein sequence of CeDGAT1 contains several conserved domains including acyl-CoA binding motif, DAG-binding site and putative catalytic active site. CeDGAT2 proteins have the conserved domains such as RGFA motifs, ER retrieval motifs (ER-DIR) and EPHS motif (Fig. 1). Phylogenetic analysis indicated that CeDGAT1 protein is grouped into the DGAT1 family while CeDGAT2s were clustered into DGAT2 family (Fig. 2). The three CeDGATs proteins do not seem to have high homology with *Arabidopsis* orthologs, but quite close to poaceae plants *Sorghum bicolor* SbDGATs and *Oryza brachyantha* ObDGATs.

### The expression pattern of *CeDGAT2-2* is consistent with oil accumulation in tuber of *C. esculentus*

Our transcriptome data showed that three *CeDGAT* genes were expressed in developing tubers with *CeDGAT2-2* much higher. To further verify their expression patterns, qRT-PCR was employed to examine their expression profiles in various tissues and different development stages of *C. esculentus* tubers*.*

**Fig. 3:**
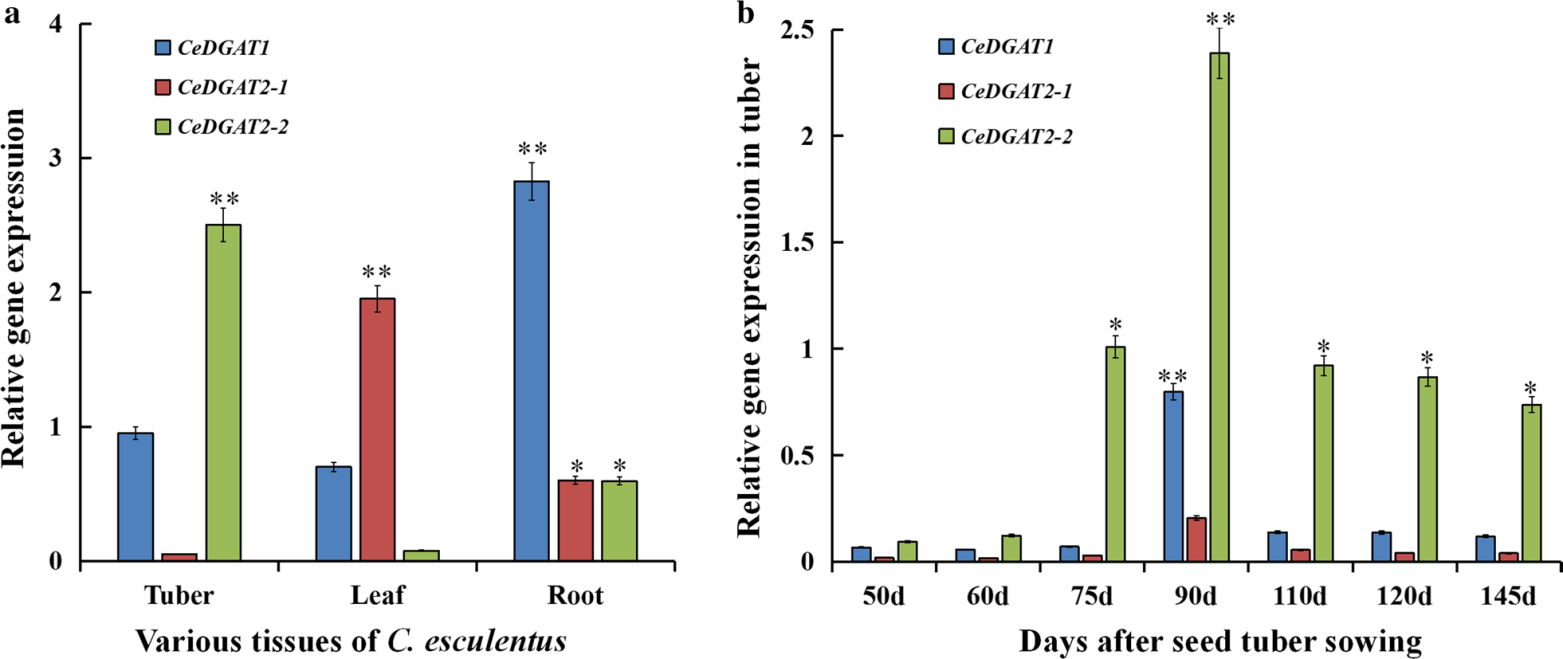
Expression patterns of three *CeDGAT* genes in various tissues (**a**) and during tuber development (**b**) of *C. esculentus.* The various tissues were collected from plants 90 days after tuber sowing. Values are the mean and standard error (*n* = 6). * and ** indicate statistically significant differences between samples at *P* < 0.05, and *P* < 0.01, respectively

The results showed that all three genes were expressed in root, leaf and tuber, but their expression levels in each tissue were significantly different (Fig. 3a). Relative transcript levels of these genes were determined by qRT-PCR using *C. esculentus 18S rRNA* as the internal control. *CeDGAT1* expression was much higher than that of *CeDGAT2-1* and *CeDGAT2-2* in roots while *CeDGAT2-1* transcript was more than that of the other two *CeDGAT* genes in leaves. Notably, *CeDGAT2-2* was predominantly expressed in tubers, with its level significantly higher that of either *CeDGAT1* or *CeDGAT2-1 *(Fig. 3b). This suggests that the three CeDGATs may function differentially in different tissues with CeDGAT2-2 as one of the key contributors to oil biosynthesis in tubers.

**Fig. 4:**
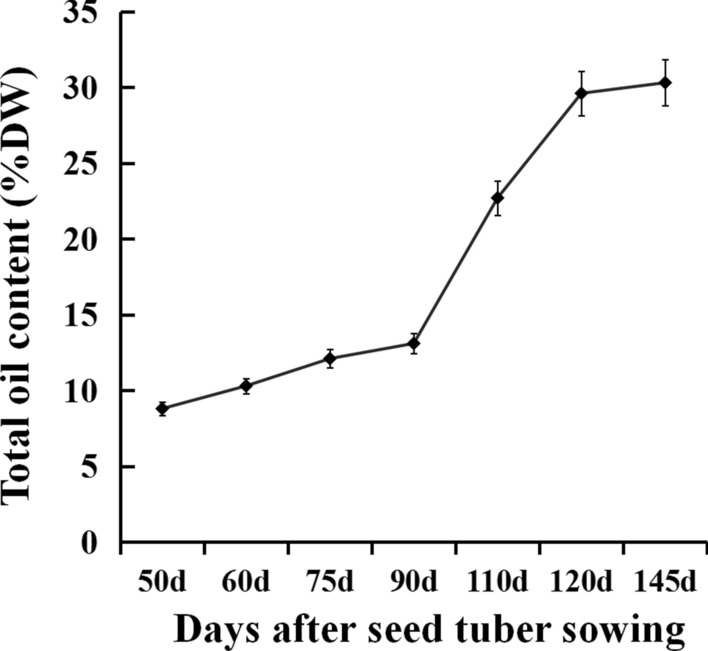
Oil accumulation during tuber development of *C. esculentus.* Total oils were extracted from samples of different developing stages of tubers and oil content was determined by the methods described in the section of “Materials and methods”. Values are means ± SE (*n* = 6)

In the present study, it was observed that the tuber development to mature needed 145 days after seed tuber sowing (DAS) in Taigu, Shanxi (E112° 28′, N37° 12′) for the variety used here. Correspondingly, the oil accumulation patterns in tubers could be briefly divided to three different stages (Fig. 4): the early stage of 50–90 DAS with low oil accumulation rate, the middle stage of 90–120 DAS with the highest oil accumulation rate, and the late stage of 120–145 DAS with slow oil accumulation rate. Therefore, the expression patterns of the three *CeDGAT* genes were examined in three different stages of the tuber development. As shown in Fig. 3b, *CeDGAT2-2* was the highest expression level at all stages of tuber development. Particularly at 90 DAS, *CeDGAT2-2* expression was 3 times as much as that of *CeDGAT1* and about 11 times as much as that of *CeDGAT2-1*, respectively. The expression pattern of *CeDGAT2-2* was a slow increased expression from 50 to 75 DAS, and then the most fast increased expression from 75 to 90 DAS, followed by a slow decline after 90 DAS. More importantly, *CeDGAT2-2* expression pattern is in accordance with oil dynamic accumulation in tubers, again suggesting that *CeDGAT2-2* might function importantly in oil synthesis and accumulation in tubers. Collectively, the highest expression level is for *CeDGAT1* in roots, *CeDGAT2-1* in leaves, and *CeDGAT2-2* in developing tubers of *C. esculentus*.

### Molecular cloning of *CeDGAT2-2* and construction of its expression vectors

As shown above, CeDGAT2-2 may be the key DGAT responsible for TAG biosynthesis in *C. esculentus* tubers. To identify such function, the ORF (954 bp) of *CeDGAT2-2* was cloned, and subsequently inserted into pYES2.0 and pBI121 vectors, respectively, to construct yeast expression vector (pYES2.0::*CeDGAT2-2*) and plant expression vector (pBI121::*CeDGAT2-2*).

**Fig. 5:**
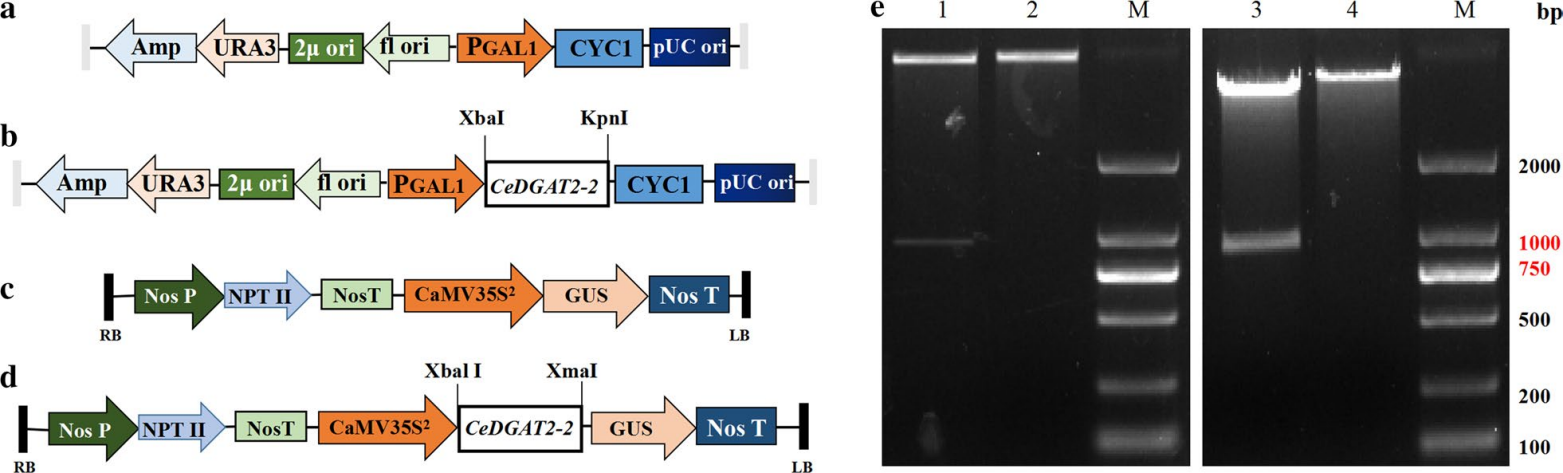
Schematic representation of *CeDGAT2-2* gene expression cassette in the vectors. **a** Empty vector without the target gene sequence was used as vector control (pYES2.0); **b** *CeDGAT2-2* expression cassette was inserted into a yeast vector pYES2.0; **c** empty vector without the target gene sequence was used as vector control (pBI121); **d** *CeDGAT2-2* expression cassette was inserted into a plant vector pBI121. Amp, ampicillin resistance gene; URA3, selection gene of yeast transformants in uracil-deficient medium; 2μ ori, maintenance and high copy replication gene in yeast; f1 ori, rescue gene of single-stranded DNA; PGAL1, promoter; CYC1, transcription termination; pUC ori, maintenance and high copy replication gene in *E. coli*; Nos P, promoter; NTP II, neomycin phosphotransferase II gene; Nos T, termination; CaMV35S^2^, promoter; GUS, report gene. **e** Gel images showing double-digested fragments of the vectors. Lane 1‚ pBI121 recombinant plasmid with *Xba*I-*Kpn*I; lane 2‚ pBI121 recombinant plasmid digested with *Xba*I; lane 3‚ pYES2.0 recombinant plasmid with *Xba*I-*Xma*I; lane 4‚ pYES2.0 recombinant plasmid digested with *Xba*I; lane M‚ 2000-bp DNA marker

As shown in Fig. 5, we inserted the *CeDGAT2-2* expression cassette at the multiple cloning sites of pYES2.0 and pBI121, respectively. The double-digestion experiment on the recombinant plasmid yielded a fragment of the same size as the target gene (Fig. 5e), indicating that the expression vectors were successfully constructed and can be used in subsequent transformation experiments.

### Overexpression of *CeDGAT2-2* functionally restores oil biosynthesis in TAG-deficient mutant of *Saccharomyces cerevisiae* H1246

For functional characterization of *CeDGAT2-2*, the yeast expression vector of this gene was constructed, and then transformed into a *S. cerevisiae* mutant strain H1246. This mutant is a quadruple yeast mutant in which four acyltransferase genes (*DGA1*,* LRO1*,* ARE1* and *ARE2*) controlling TAG biosynthesis was interrupted, showing no TAG biosynthesis in cells [36].

**Fig. 6:**
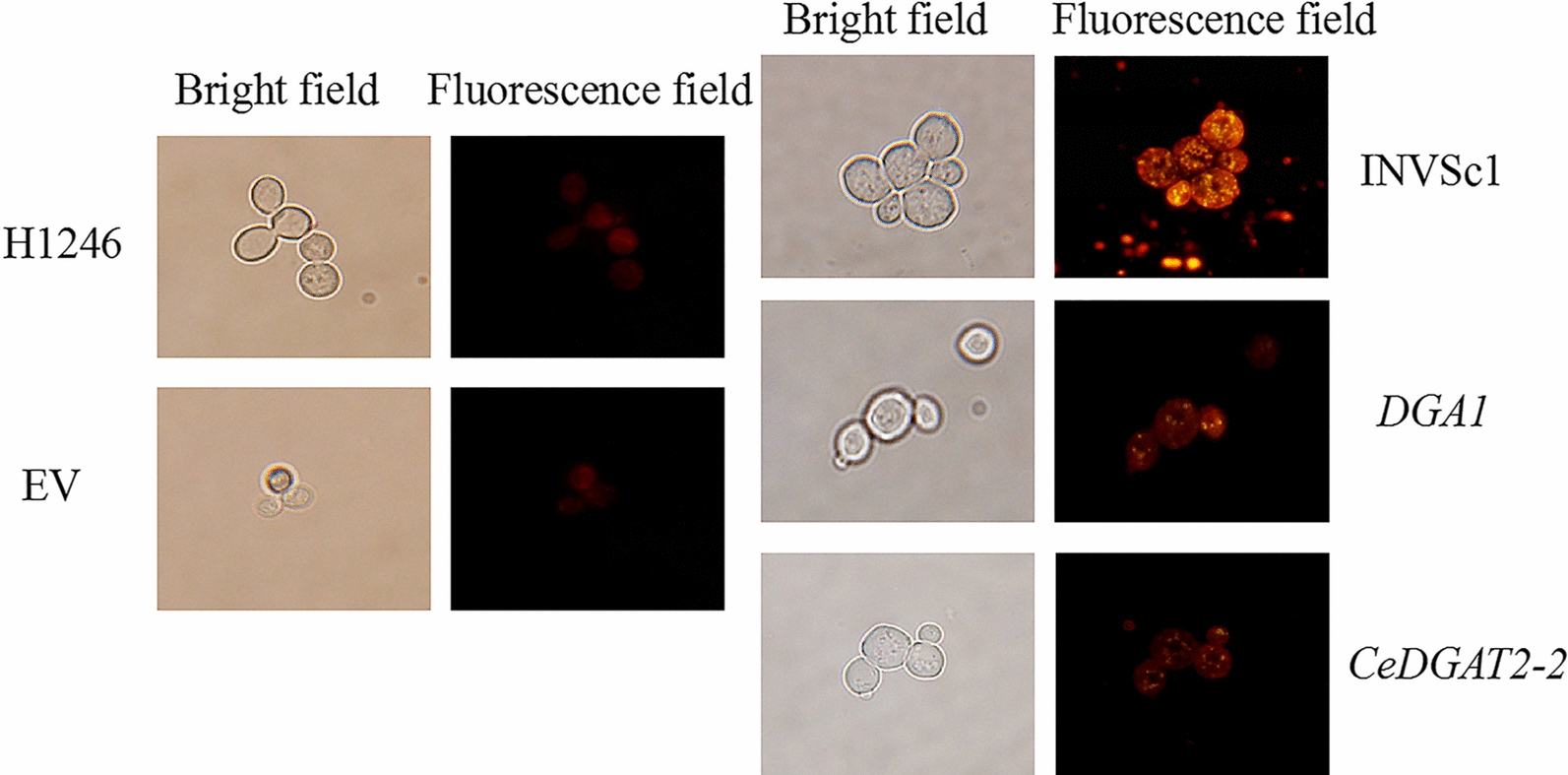
Fluorescence microscopy of various yeast strains stained with Nile Red. H1246‚ TAG-deficient quadruple mutant strain of *C. cerevisiae*; EV‚ H1246 harboring the empty plasmid pYES2.0; INVSc1‚ wild-type yeast strains; *DGA1‚* H1246 expressing yeast *ScDGA1* gene; *CeDGAT2-2*‚ H1246 expressing *CeDGAT2-2* gene

The H1246 expressing the empty vector (EV) and the H1246 expressing yeast *ScDGA1* gene were used as the negative and positive control, respectively. Nile Red staining was used to identify lipid droplet formation. Nile red dye can excite yellow fluorescence under blue light when combined with neutral lipid. In this study, no fluorescence was observed in the transformed H1246 cells with empty vector, as similar to the control H1246, indicating no formation of oil bodies in these yeast cells. However, a large number of lipid droplets were produced in wild-type yeast INVSc1 and the mutant H1246 expressing yeast *ScDGA1*. And thus, TAG synthesis was restored in the mutant strain H1246 transformed by the *ScDGA1* gene. Similarly, H1246 cells expressing *CeDGAT2-2* displayed strong fluorescence signal, showing that CeDGAT2-2 can restore the formation of oil bodies like the case of yeast ScDGA1 (Fig. 6).

**Fig. 7:**
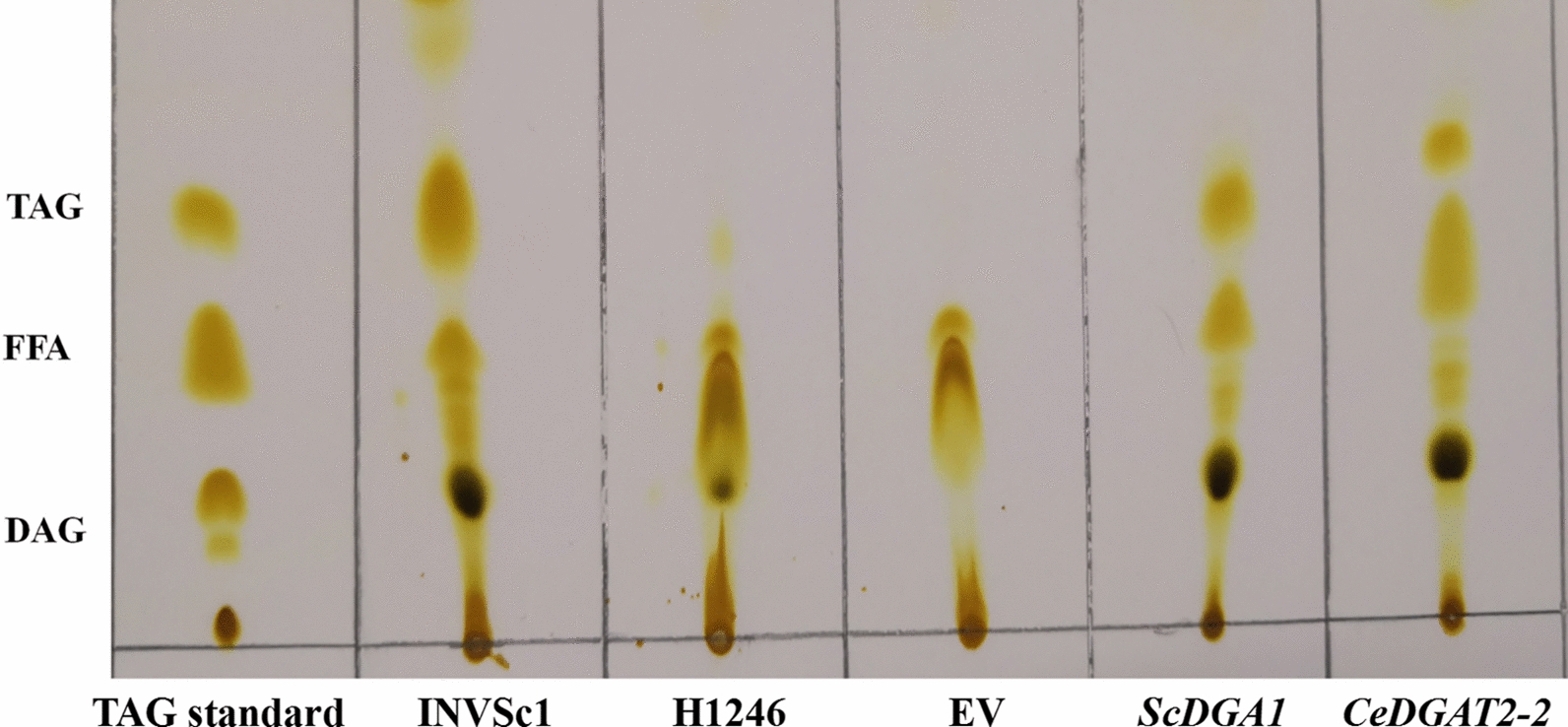
Thin-layer chromatography (TLC) of the neutral lipids from various yeast strains. TAG standard; INVSc1‚ wild-type yeast strains; H1246‚ TAG-deficient quadruple mutant strain H1246 of *C. cerevisiae*; EV‚ H1246 harboring the empty plasmid *pYES2.0*; *ScDGA1*‚ H1246 expressing *ScDGA1* gene; *CeDGAT2-2*‚ H1246 expressing *CeDGAT2-2* gene

Neutral lipids extracted from H1246 and the transgenic yeast cells were analyzed by thin-layer chromatography (TLC) (Fig. 7). As expected, almost no TAG band was detected in the two negative controls, the mutant H1246 cells and H1246 harboring an empty vector pYES2.0. On the contrary, a prominent TAG band appeared in the H1246 cells expressing *CeDGAT2-2* or *ScDGA1* and the wild-type positive controls, showing that either *CeDGAT2-2* or *ScDGA1* expression could complement the mutation and successfully rescue the deficiency of TAG synthesis in mutant H1246.

**Fig. 8:**
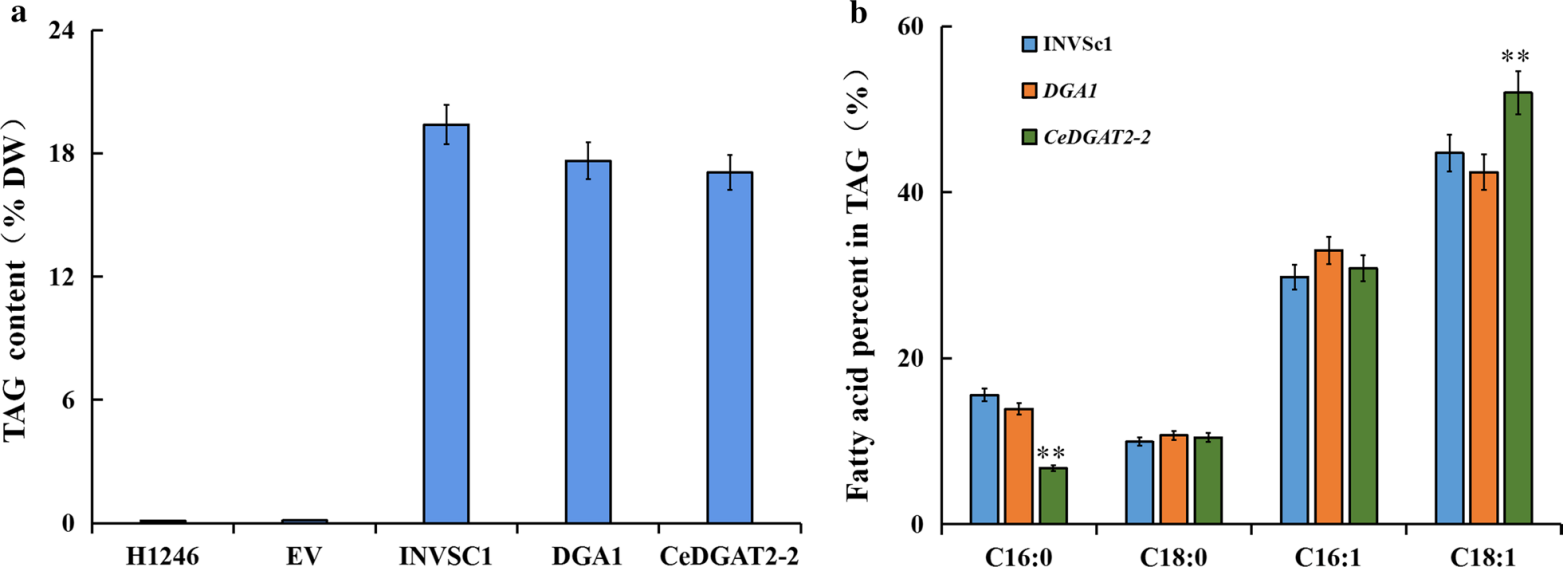
TAG content (**a**) and fatty acid profiles (**b**) in various yeast strains cultured without feeding of exogenous fatty acids. Fatty acid content was expressed as percentage of cell dry weight. Each value is the mean ± SE of six biological replicates.* and ** indicate statistically significant differences between samples (*t*-test) at *P* < 0.05, and *P* < 0.01, respectively

Further quantitative analysis of the level of TAG demonstrated that the overexpression of *CeDGAT2-2* or *ScDGA1* triggered significant production of TAG in H1246 cells, but TAG levels in the two transgenic yeasts were not as much as in wild-type INVSc1 (Fig. 8a). In order to elucidate the FA profiles in TAGs produced by CeDGAT2-2 or ScDGA1 in the yeast samples, the TAG spots on the TLC plates were scraped off, and then lipid were recovered to be transesterified for GC analysis (Fig. 8b). In the wild-type yeast, major fatty acids are C18:1 (37.73%), C16:1 (36.80%), C16:0 (15.57%) and C18:0 (9.93%). The content of each of these four fatty acids in the *ScDGA1*-transgenic yeast was similar as in the wild-type control. However, in the CeDGAT2-2 transgenic H1246 cells, C18:1 was significantly increased while C16:0 was decreased compared to the positive controls, showing that CeDGAT2-2 might have preference for unsaturated fatty acid, especially for C18:1.

To test the substrate specificity of enzyme encoded by *CeDGAT2-2*, the yeast cells were cultured with and without presence of exogenous fatty acids in the medium. The major fatty acids in tuber oil of *C. esculentus* are C18:1 (60–75%), C18:2 (~ 12%), and C16:0 (~ 12%). And thus, these three fatty acids (C18:1, C18:2 and C16:0) were added into the medium, respectively. Fatty acid composition in TAG synthesized in these yeast cells was examined by GC analysis. As shown in Table 1, compared to the yeast without adding any of the three fatty acids, fatty acid percentage was not significantly changed in the wild-type and H1246 expressing *ScDGA1* when the three fatty acids were added, respectively. For H1246 cells expressing *CeDGAT2-2*, C18:1 and C18:2 levels were increased with the former much higher than the latter when C18:1 and C18:2 were added, respectively, while C16:0 has no significant change in presence of exogenous C16:0. This feeding assay again evidences that CeDGAT2-2 have substrate selection for unsaturated fatty acids, particularly for C18:1.

**Table 1:** Fatty acid profiles in various yeast strains cultured with feeding of exogenous fatty acids

Strains	Fatty acid composition (% of total FAs)				
	C16:0	C16:1	C18:0	C18:1	C18:2
No FA adding					
Wild-type	15.57 ± 0.64	36.80 ± 1.53	9.93 ± 0.67	37.73 ± 1.08	
CeDGAT2-2	11.75 ± 1.23*	32.83 ± 1.64	10.44 ± 1.08	44.98 ± 0.95*	
C16:0 adding					
Wild-type	15.93 ± 1.02	37.66 ± 0.81	9.78 ± 1.06	36.63 ± 1.34	
CeDGAT2-2	14.06 ± 0.62*	32.03 ± 0.81	10.31 ± 1.27	43.60 ± 0.57*	
C18:1 adding					
Wild-type	15.97 ± 0.99	35.99 ± 0.43	9.01 ± 0.68	39.03 ± 1.38	
CeDGAT2-2	9.82 ± 0.89*	30.49 ± 0.73	9.83 ± 0.12	49.86 ± 1.08**	
C18:2 adding					
Wild-type	14.98 ± 1.28	33.67 ± 1.93	10.17 ± 0.84	35.71 ± 0.72	5.47 ± 0.09
CeDGAT2-2	6.54 ± 0.75*	30.98 ± 1.22*	8.52 ± 0.85	40.21 ± 1.48*	13.75 ± 0.81**

Fatty acid content was expressed as percentage of total FAs. Each value is the mean ± SE of six biological replicates.* and ** indicate statistically significant differences between samples (*t*-test) at *P* < *0.05,* and *P* < *0.01,* respectively

### Overexpression of *CeDGAT2-2* significantly increases oil and oleic acid accumulation in tobacco leaves

**Fig. 9:**
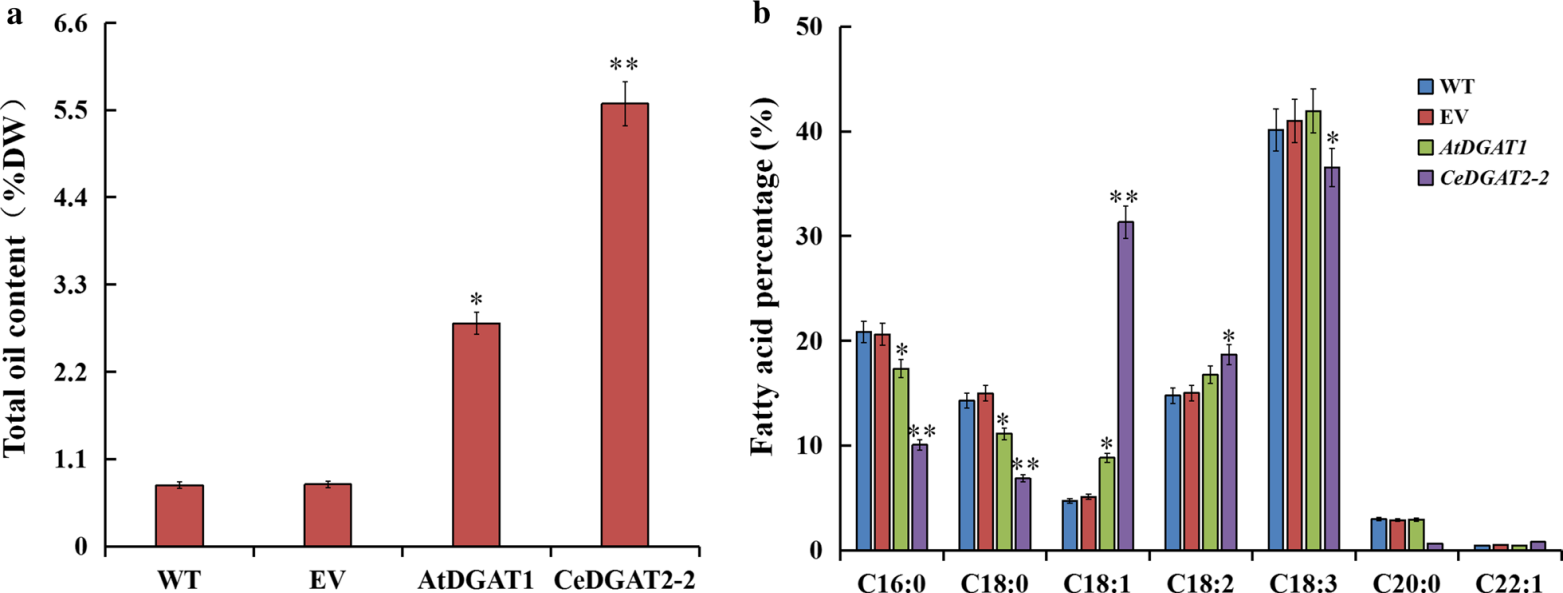
Total oil content (**a**) and fatty acid profiles (**b**) in *N. tabacum* leaves expressing *CeDGAT2-2* and *AtDGAT1* genes, respectively. WT, wild-type tobacco plant; EV, the tobacco plant expressing empty vector; AtDGAT1, the tobacco plant expressing *AtDGAT1* gene; CeDGAT2-2, the tobacco plant expressing *CeDGAT2-2* gene. Total oil content was expressed as percentage of leaf dry weight. The top fully expanded leaf from tobacco plants at floral transition stage was sampled. Lipids were extracted from the leaf samples and oil content was determined by the method described in the section of “Materials and methods”. For fatty acid profiling, lipids were transesterified, followed by gas chromatography (GC) analysis. Each value is the mean ± SE of six biological replicates. * and ** indicate statistically significant difference compared with the control (*t*-test) at *P* < 0.05 and *P* < 0.01, respectively

To evaluate the potential of *CeDGAT2-2* in plant lipid improvement, particularly in vegetative organs to increase both total oil and desirable oleic acid accumulation, plant constitutive expression vector of *CeDGAT2-2* was developed and transformed into tobacco by *Agrobacterium*-mediated leaf disc transformation. *Arabidopsis* AtDGAT1 was used as the positive control because AtDGAT1 was already identified to increase oil content in tobacco leaves. Compared with wild-type tobacco, the total lipid content of transgenic plants increased significantly (Fig. 9a). Transgenic plants overexpressing *CeDGAT2-2* gene had higher oil content in leaves, which enhanced by 7.15 times compared to the wild type, and 1.7 times compared to *AtDGAT1* transgenic leaves, respectively, indicating that the enzymatic activity of CeDGAT2-2 is higher than that of AtDGAT1 for TAG biosynthesis in the vegetable tissues when they are heterogeneously expressed in tobacco plants.

Analysis of fatty acid profiles in leaf oil (Fig. 9b) revealed that there was a slight difference between *AtDGAT1*-overexpressed plants and the control plants. Surprisingly, compared to the control plants (wild-type and empty-vector transgenics), *CeDGAT2-2* overexpression resulted in the significant alteration of fatty acid percentage in leaf oil, showing that levels of unsaturated fatty acids (C18:1, C18:2) were increased while contents of saturated fatty acids (C16:0 and C18:0) were accordingly decreased. In particular, the C18:1 content increased from 5.1% in the wild type to 31.33% in the *CeDGAT2-2*-overexpressed tobacco leaf oil. Notably, CeDGAT2-2 exhibited substrate selectivity towards unsaturated fatty acids, with much preference for C18:1. Collectively, these results evidence that CeDGAT2-2 is one of main contributors to oil biosynthesis*,* particularly for high accumulation of oleic acid (60–75%) in tubers of *C. esculentus*. More importantly, CeDGAT2-2 can be used in gene engineering high-biomass plants to increase both oil content and desirable oleic acid accumulation in the vegetable tissues, which could help meet our growing demand for plant oils.

### Overexpression of *CeDGAT2-2* leads to no significant negative effects on other agronomic traits of tobacco plants

**Fig. 10:**
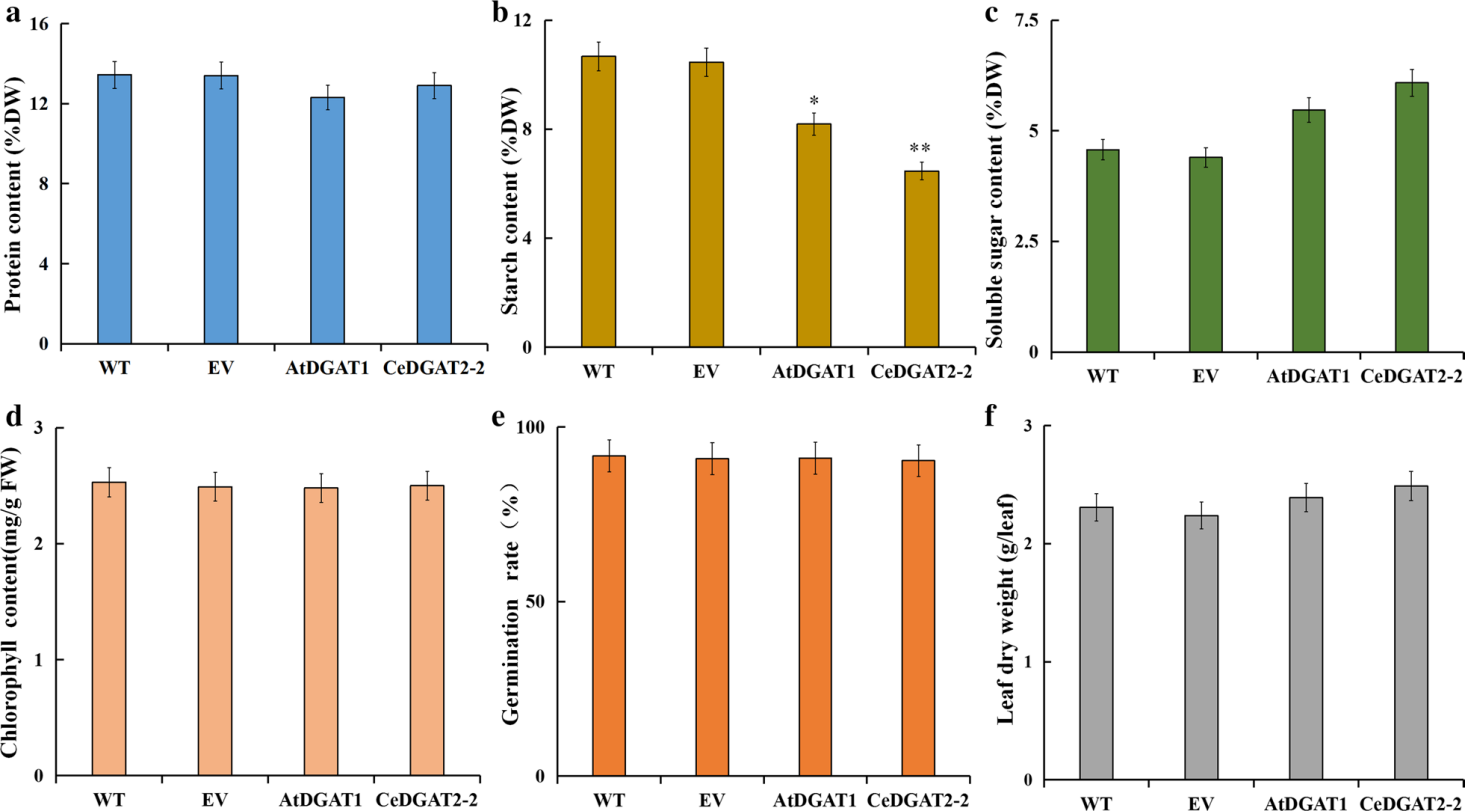
Other agronomic traits in *N. tabacum* expressing *CeDGAT2-2* or *AtDGAT1* gene, respectively. **a** Total protein content; **b** total starch content; **c **total soluble sugar content; **d** chlorophyll content; **e** germination rate; **f** leaf biomass (dry weight). WT, wild-type tobacco plant; EV, the empty-vector transgenic tobacco plant; CeDGAT2-2, the *CeDGAT2-2-*transgenic tobacco plant; AtDGAT1, the *AtDGAT1-*transgenic tobacco plant. The top fully expanded leaf from tobacco plants at floral transition stage was sampled and divided into a number of batches for different analysis. Each value represents the mean ± SE of six biological replicates. Asterisks indicate statistically significant difference compared with the control (*t*-test) at * *P* < 0.05 and ** *P* < 0.01, respectively

To evaluate whether *CeDGAT2-2* overexpression affects other agronomic traits, we further examined levels of soluble sugar, starch, protein and chlorophyll in the leaves as well as leaf dry mass and seed germination rate of the transgenic tobacco lines. As shown in Fig. 10a, protein content was a little difference between the control and the transgenic plants of either *AtDGAT1* or *CeDGAT2-2.* Starch content was reduced from 10.67% in the control to 8.18% in *AtDGAT1*-transgenic and 6.46% in *CeDGAT2-2* transgenic plants, respectively (Fig. 10b). For soluble sugar content, 1.68% and 1.07% of increase were detected in *CeDGAT2-2* and *AtDGAT1* transgenic plants, respectively, compared to the control (Fig. 10c). No obvious changes were detected for total chlorophyll content (Fig. 10d), and seed germination rate (Fig. 10e) among the samples. For leaf biomass, there was also no significant difference between the controls and *AtDGAT1* transgenics, but a small increase in the *CeDGAT2-*2 transgenic plants (Fig. 10f). Taken together, these data demonstrate that overexpression of *CeDGAT2-2* in tobacco can significantly increase carbon source into lipid biosynthesis pathway, leading to great enhancement of total leaf oil and oleic acid accumulation without negative effect on photosynthesis, plant growth, seed germination and other agronomic traits. High leaf oils enriched with the desirable oleic acid are favorable for their utilization as high-quality oil and biodiesel.

## Discussion

### CeDGAT2-2 is a key player functioning crucially for high levels of oleic acid and oil storage in underground tubers of *C. esculentus*

TAGs are the predominant storage form of energy in eukaryotic cells. The final acylation of TAG synthesis is catalyzed by DGAT, which is recognized as the major flux control step in oil biosynthesis. DGAT is a key target focused by many researchers because it is an enzyme unique to TAG biosynthesis in plants. To date, numerous DGATs derived from seeds rich in oil have been well characterized [21–26]. However, just a few DGATs come from oil-enriched vegetative plant tissues were investigated [13, 30, 31].

In this study, three *DGAT* genes (*CeDGAT1*, *CeDGAT2-1 and CeDGAT2-2*) were identified from *C*. *esculentus*, a special oil crop accumulating high level of oils in its underground tubers [34], which is significantly different from common underground crops like potato and cassava enriching large amounts of storage carbohydrates in their underground parts. These three *CeDGAT* gene expressions were detected to be somewhat tissue-specific patterns, with *CeDGAT1* highly expressed in roots, *CeDGAT2-1* largely in leaves, and *CeDGAT2-2* much abundantly in developing tubers (Fig. 3a), suggesting that these CeDGAT enzymes may function differentially in oil biosynthesis in different tissues. Especially, *CeDGAT2-2* expression dynamics (Fig. 3b) was highly correlated with oil accumulation pattern during developing tubers (Fig. 4), showing that CeDGAT2-2 may play much important roles for oil accumulation in *C*. *esculentus* tubers.

Functional characterization assay in yeast evidenced that CeDGAT2-2 is a functional DGAT enzyme because overexpression of *CeDGAT2-2* resulted in restoration of TAG synthesis in TAG-deficient yeast mutant H1246 (Figs. 6, 7). Moreover, CeDGAT2-2 exhibited a strong substrate specificity for oleic acid (18:1), indicated by higher level of TAG produced when *CeDGAT2-2* transgenic yeast was cultured in the medium with addition of exogenous oleic acid (Table 1). Such enzyme features of CeDGAT2-2 combined with its expression profile during tuber development clearly demonstrate that CeDGAT2-2 is the main contributor for high level of oleic acid and total oil accumulation in *C*. *esculentus* tubers.

Unlike CeDGAT2-2 described here, several DGAT2s derived from plant seeds highly accumulating non-edible oils were identified to have a substrate preference for UFAs and function substantially for TAG synthesis enriched with UFAs. For example, RcDGAT2 from castor bean (*Ricinus communis*) could effectively incorporate ricinoleic acid (18:1-OH) into TAGs [24]. VgDGAT2 derived from *Vernonia galamensis* seeds had high substrate selectivity for vernolic acids (18:1-OE) [21, 25]. Nevertheless, further study needs to investigate the mechanism responsible for such difference between CeDGAT2-2 and other DGAT2s from non-edible oil seeds.

It is also worth noting that DGAT1 is believed to be a key enzyme in most oil-rich seeds and some non-seed oil tissues for TAG synthesis enriched with normal FAs [37]. For instance, DGAT1 played a vital role in oil biosynthesis and accumulation in seeds of *Arabidopsis* [38], soybean [22] and rapeseed [39], as well as in mesocarp tissues of avocado [40], and olive [41]. Moreover, both DGAT1 and DGAT2 were also detected to be the main contributors to TAG synthesis in some plants [26]. All these suggest that different DGAT1s and DGAT2s might be active differentially in different plant species and even in different tissues or developing stages of the same plant, with some cases showing redundance or overlapping functions.

### *CeDGAT2-2* is a desirable target gene in metabolic engineering to enhance oil accumulation in plant vegetative tissues for biofuel oil production

As the rate-committed enzyme for TAG biosynthesis, DGAT has been widely used for the core target of genetic manipulation to improve oil yield and fatty acid profiles in both seeds and non-seed plant tissues. For example, seed-specific overexpression of *AtDGAT1* increased seed weight and oil content of *Arabidopsis* [38] and *Brassica juncea* [42]. Moreover, *AtDGAT1* expression in tobacco leaves led to leafy oil increased up to 5.8–6.0% of DW [12], and increased oil content by 20–30% in seeds and 1.5- to 2.0-fold in leaves of *Jatropha curcas* [43]. A fungal *DGAT2* overexpression resulted in oil enhancement in soybean [44] and maize [45].

Ectopic overexpression of DGAT also resulted in alternation of FA profile in the target tissues. For example, the overexpression of *JcDGAT1* in *Arabidopsis* resulted in a significant decrease in oleic acid (C18:1) and an increase in linolenic acid (C18:3) [46], and the transgenic expression of *S. indicum* *DGAT1* in *Arabidopsis* led to an increase in eicosenoic acid (C20:1) and a reduction in oleic acid (C18:1) in seed oil [47]. Overexpression of *VgDGAT1a* derived from *V. galamensis* increased tobacco leafy oil by 3.5- to 5.0-fold compared to the control, accompanied by a significant enhancement of linoleic acid and notable reduction of ɑ-linolenic acid [31]. In tobacco leaves expressing *AtDGAT*, oleic acid (18:1) increased from 1.5% in WT tobacco to 20% around in transgenic lines, followed by a significant decrease in linolenic acid (18:3) (from 67% down to ~ 35%) and the unchanged content of linoleic acid (18:2) [12]. *AtDGAT2* overexpression also changed the fatty acid composition in the transgenic leaves [48]. Another case was that *AtDGAT1* expression increased oil content in tobacco leaves, but no alteration of FA profile [30].

All these above indicated that different DGAT members or source-different DGATs display diverse impact and efficiency in this regard. In the present study, we aim to improve leafy oil yield and FA composition in tobacco by metabolic engineering so as to develop tobacco as an ‘energy platform crop’. Following the functional characterization of CeDGAT2-2, the transgenic tobacco plants overexpressing *CeDGAT2-2* were developed, and finally their phenotypes were examined. Our data demonstrated that transgenic tobacco plants overexpressing *CeDGAT2-2* increased leafy oils by 7.15 times compared to the wild type (Fig. 9a). This oil level in the leaves was 1.7 times higher than that in *AtDGAT1*-transgenic leaves (the positive control). In particular, oleic acid (18:1) content was increased from 5.1% in the wild type to 31.33% of leafy oil in the *CeDGAT2-2* transgenic tobacco (Fig. 9b), accompanied by large reduction of polyunsaturated fatty acids (PUFAs, 18:2, 18:3). However, oleic acid content just had a small increase in *AtDGAT1*-transgenic leaves. Furthermore, the transgenic tobacco plants exhibited no negative phenotypes of other agronomic traits (Fig. 10) including the seed yield, seed germination, and other morphological and developmental features.

In the context of developing tobacco biomass oil into diesel fuel, an observed dramatic shift of TAG FA profiles to a higher concentration of oleic acid and lower PUFAs in *CeDAGT2-2* tobacco is highly desirable. The shift to a higher content of oleic acid and lower proportion of PUFAs maximizes the fuel characteristic of the oil by enhancing the oxidation stability and improving the cold flow properties of biodiesel [49]. Such high level of oleic acid in the transgenic leave might be related with the strong substrate specificity of CeDGAT2-2 for oleic acid. An explanation of these findings could be the limited availability of desaturase, which leads to the accumulation of more mono- and di-unsaturated FA when the total amount of FA increases [50].

Previous reports demonstrated that it is feasible to develop transgenic plants to produce oil in leaves. For example, several fold increases in the accumulation of oil (TAG) were achieved in *Arabidopsis thaliana*, sugarcane, sorghum and tobacco through the overexpression of lipid related transcription factors and biosynthetic genes. Further increases in TAG content can also be achieved through silencing of competing pathways such as starch synthesis or TAG degradation, and combined with overexpression of more genes responsible for carbon supply and partitioning [51]. A newly characterized type-II DGAT (*CeDGAT2-2*) derived from *C. esculentus* in this study would be the ideal DGAT for this integrate metabolic engineering to develop “new type of oil crops” which can largely accumulate oils in high-biomass nutrient organs enriched with better FA profiles for sustainable production of high-quality edible or industrial oils.

## Conclusion

The present study reveals the physicochemical property and expression profiles of three *DGAT* genes (*CeDGAT1, CeDGAT2-1* and *CeDGAT2-2*) identified from *C. esculentus,* a special oil crop uniquely accumulating high level of oleic acid-enriched oil in its underground tubers. *CeDGAT2-2* expression pattern is well coordinated with oil dynamic accumulation during tuber development. Functional characterization using yeast essay and transgenic tobacco demonstrate that CeDGAT2-2 has a high enzymatic activity to catalyze TAG biosynthesis, and also a strong specificity for oleic acid-containing substrates, which is distinguished from other DGAT2s responsible for selective incorporation of unusual fatty acids into TAGs in several source oil plants. CeDGAT2-2 is a key contributor for storage oil accumulation in *C. esculentus* tubers. Remarkably, overexpression of *CeDGAT2-2* alone significantly increase total oil and oleic acid accumulation in tobacco leaves and such effect is much higher than the expression of *AtDGAT1* derived from *Arabidopsis* seeds. Moreover, *CeDGAT2-2* transgenic tobacco exhibits no compensating plant growth and seed traits. As a desirable target gene, *CeDGAT2-2* overexpression in combination with modification on other related genes could enable the engineered tobacco plant to enrich much large amount of storage oil in non-seed biomass for sustainable production of biofuel oils. These findings expand our understanding about the function of DGAT in plant vegetative tissues, providing new knowledge for further optimization of metabolic assembly of oil-enriching pathway in plant for greater oil yielding.

## Materials and methods

### Plant materials

*Cyperus esculentus* (var. Jinnong-01) was planted in Taigu County (E112° 28′–113° 01′, N37° 12′–37° 3′2), Shanxi Province, China. Tubers at different development stages were collected. After washing with water, the tubers were dewatered by filter papers and stored at − 80 °C for late use.

Tobacco seedlings in a 1/2 MS (Murashige and Skoog) (30 g/L sucrose, 7 g/L agar, pH 5.8) were grown in a growth chamber under fluorescent light (~ 130 μmol m^−2^ s^−1^) and with the following conditions: 16/8 h light/dark, 26 ± 2 °C, and 40–60% humidity.

Yeast strains were maintained in 2% (w/v) yeast extract, 1% (w/v) peptone and 2% (w/v) dextrose (YPD) medium at 30 ± 0.5 °C.

### Bioinformatics analysis of proteins encoded by *CeDGAT* genes in* C. esculentus*

The conserved domain database (CDD) in NCBI (http://www.ncbi.nlm.nih.gov/Strucdd/cdd.shtml) and MEME (http://meme-suite.org/tools/meme) were employed to identify the conserved domains of three CeDGAT proteins. The Protparam was used to analyze the physical and chemical properties of amino acid composition, relative molecular weight, theoretical and electrical points and atomic composition. The TMHMM (http://www.cbs.dtu.dk/services/TMHMM/) was used to detect cross-membrane structures. Plant-mPLoc (http://www.csbio.sjtu.edu.cn/bioinf/plant-multi/predicts) was employed to predict subcellular location. The secondary structure of the protein was predicted using PBIL LYON-GERLAND. Multiple sequence alignment was conducted by Genedoc. Phylogenetic analyses were carried out using MEGA 7.0. Phylogenetic trees were constructed by neighbor-joining method with a bootstrap of 1000 replicates.

### Expression profile of *CeDGAT* genes in *C. esculentus*

Total RNA was isolated using *Transgen* plantTrazol extraction kit. The obtained pure RNA was reversed to the first cDNA using the ABM 5 × ALL-IN-ONE RT Master Mix kit (Canada). The obtained cDNA samples were stored at − 80 °C for later use.

qRT-PCR was performed on the CFX96 Real-Time PCR Detection System (BIO-RAD, Hercules, CA, USA) using the SYBR Premix Ex Taq II (Takara) and a program with initial 95 °C for 10 min followed by 40 cycles of 95 °C for 15 s, 60 °C for 30 s, 72 °C for 30 s. Primers for qPCR are shown in Table S1. The 2^−∆∆Ct^ calculation was used to determine the relative expression. The expression values of *CeDGAT* genes were quantified by using *C. esculentus 18S rRNA* as an internal standard.

### *CeDGAT2-2* gene cloning and vector construction

Full-length cDNA of *CeDGAT2-2* and *AtDGAT1* (AT2G19450) were amplified with specific primers (Additional file 3). The cDNAs were subsequently used for amplification of ORF by RT-PCR using *EasyPfu* DNA Polymerase. Two amplicons were cloned into pGEM T-easy vector (Promega, Madison, WI, USA), respectively, and their nucleotide sequences were determined by sequencing.

The ORF of *CeDGAT2-2* was separately amplified from their corresponding clone vectors using gene-specific primers with *Xba*l (5′-end) and *Xma*I (3′-end) sites (pBI121::*CeDGAT2-2-*F: 5′-GCTCTAGAATGGGAAACAAGGAAACAATGGA-3′ and pBI121::*CeDGAT2-2-*R: 5′-CCCCCGGGCTACAACACTCTCAGTTGAAGAT-3′). *CeDGAT2-2* ORF was then inserted behind the CaMV 35S promoter in the pBI121 vector. These vectors containing *CeDGAT2-2* were then transformed into *Agrobacterium tumefaciens* GV3101 by the freeze–thaw method, which were consequently used for *N. tabacum* plant transformation.

The ORF of *CeDGAT2-2* was amplified from clone vectors using primers containing *Kpn*I (5′-end) and *Xba*I (3′-end) sites (pYES2.0::*CeDGAT2-2-*F: 5′-GGGGTACCATGGGAAACAAGGAAACAATGGA-3′ and pYES2.0::*CeDGAT2-2-*R: 5′-GCTCTAGACTACAACACTCTCAGTTGAAGAT-3′). The ORF of *CeDGAT2-2* was then cloned into the yeast expression vector *pYES2.0* under control of the inducible promoter GAL1.

### Heterologous expression in the *S. cerevisiae* mutant H1246

The yeast expression vector of the target gene was transformed into the *Saccharomyces cerevisiae* TAG-deficient mutant strain H1246 [36] using the polyethylene glycol/lithium acetate method. H1246 cells harboring the yeast *DGA1* gene encoding *DGAT2* and the empty pYES2.0 vector were used as the positive and negative controls, respectively. Transformants were screened on yeast synthetic complete medium lacking uracil (SC-Ura). After induction with galactose for 2 days, H1246 cells were collected by centrifugation (5000*g* for 5 min), washed three times with distilled water, and dried for lipid extraction and analysis. Palmitic acid (C16:0), oleic acid (C18:1) and linoleic acid (C18:2) were dissolved in Tween 80 and added into the medium with final concentration of each fatty acid of 1 mM to determine the fatty acid preference of CeDGAT2-2 with INVSc1 as the control. Total lipids were extracted and then dissolved in chloroform. TAG were separated in neutral lipid by thin-layer chromatography (TLC) in the developing agent of hexane/diethyl ether/acetic acid (80:20:1, v/v/v).

H1246 cells were harvested by centrifugation (3000 *g* for 5 min), and resuspended in distilled water to OD_600_ = 0.5. After that, yeast cells were stained with Nile Red (at a working concentration of 0.5 mg/mL). Stained cells were visualized under fluorescence microscope (BX53, OLYMPUS) using blue fluorescence.

### Tobacco plant transformation

The *CeDGAT2-2* gene expression vector was transformed into leaf discs of 30-day sterile tobacco seedlings using *Agrobacterium*-mediated transformation [52]. Transgenic shoots were selected on kanamycin (50 mg/ml) and were rooted on half-strength MS medium containing kanamycin (50 mg/ml). The rooted shoots were transferred to potting mixture and hardened plants were established in a growth chamber. The homozygous transgenic plants were obtained by screening positive transgenic tobacco plants. Seven-week-old tobacco leaves were collected for subsequent analysis and testing.

### Lipid analysis

Freeze-dried leaf or yeast cell samples were ground into powder and 50 mg powder was used for total lipid extraction by following method described by Li et al. [21]. Tri 17:0-TAG in chloroform was added into sample as the internal control. The samples were homogenized in chloroform and methanol (v:v = 2:1) containing 0.001% BHT, and then spun for a few minutes for phase separation. The lower clear liquid phase was transferred into a clean glass tube, and the sample was dried by N_2_ flow. After that, concentrated sulfuric acid in methanol was added into the tube for FA esterification at 80 °C for 2 h. Finally, n-hexane was added into the esterified samples with well mixture. The upper layer containing the FA methyl esters (FAME) was transferred into GC auto-sampler vials. The samples were measured by gas chromatography (Agilent7890B, 100 m × 0.250 mm × 0.2 μm HP-88, FFAP (free fatty acid phase) column, flame ionization detector (Agilent Technologies, Santa Clara, CA, USA). The inlet temperature and oven start temperature were set to 250 °C and 140 °C, respectively. The column was programmed with an initial temperature of 140 °C for 5 min, then increased at 2 °C/min to 250 °C for 12.5 min. Helium was used as the carrier gas, with a flow rate of 10 ml/min.

### Soluble sugar, protein and starch analysis

The soluble sugar, protein and starch were determined using Plant Soluble Sugar Content Detection Kit, Bradford protein assay kits and Starch Content Detection Kit (Beijing Solarbio Science & Technology Co., Ltd., Beijing, China), respectively. The colorimetry of samples was examined with enzymatic-reader at 620 nm and 595 nm (YT-MB96S Microplate Reader, China).

### Data analysis

All experiments were independently performed at least in six replicates. The statistical analysis of data was carried out using the program of the Statistical Package for the Social Sciences (SPSS version 22.0).

## Supplementary Information

**Additional file 1.** Three *CeDGAT* gene cDNA sequences.

**Additional file 2.** Physical and chemical properties of CeDGAT protein analysis in *Cyperus esculentus* L*.*

**Additional file 3.** Primers used in this study.

## Data Availability

The datasets supporting the conclusions of this article are included within the article and its additional files.

## Abbreviations

TAG: Triacylglycerol
DGAT: Diacylglycerol acyltransferase
DAG: Diacylglycerol
DAS: Days after seed tuber sowing
TFA: Total fatty acid
