# Correction to: Ectopic overexpression of a type-II DGAT (CeDGAT2-2) derived from oil-rich tuber of *Cyperus esculentus* enhances accumulation of oil and oleic acid in tobacco leaves

**DOI:** 10.1186/s13068-021-01990-2

**Published:** 2021-06-16

**Authors:** Yu Gao, Yan Sun, Huiling Gao, Ying Chen, Xiaoqing Wang, Jinai Xue, Xiaoyun Jia, Runzhi Li

**Affiliations:** 1 College of Agriculture, Institute of Molecular Agriculture and Bioenergy, Shanxi Agricultural University, Taigu, 030801 Shanxi China; 2 College of Life Sciences, Shanxi Agricultural University, Taigu, 030801 Shanxi China

## Correction to: Biotechnol Biofuels (2021) 14:76 https://doi.org/10.1186/s13068-021–01928-8

Following publication of the original article [1], it was brought to our attention that we missed and failed to cite a related paper [2] (Liu et al. Plant Cell Physiol, 2020; 61:118–129) that was published a short while before. Liu et al. [2] described the identification and molecular characterization of *CeDGAT1* and *CeDGAT2a/b* (*CeDGAT2-1* and *CeDGAT2-2* in [1]) from *Cyperus esculentus*. Our study [1] obtained the same results in gene expression patterns and confirmed that CeDGAT2b exhibited DGAT activity and is therefore likely the major contributor to tuber oil biosynthesis. Extending these results, our experiments demonstrated that CeDAGT2-2 had a very strong substrate specificity for oleic acid in transgenic yeast cells and tobacco leaves. More significantly, overexpression of *CeDGAT2-2* in tobacco resulted in a high level of oleic acid and total oil accumulation, and the transgenic plants exhibited no negative impact on other agronomic traits. Therefore, our finding demonstrates that *CeDGAT2-2* is the desirable target gene in metabolic engineering to enrich oil and value-added lipids in high-biomass plants. We sincerely apologize for our missing of the related work of Liu et al. [2].
