# Supplementary material for: Ectopic overexpression of a type-II DGAT (CeDGAT2-2) derived from oil-rich tuber of Cyperus esculentus enhances accumulation of oil and oleic acid in tobacco leaves
Source: Biotechnol Biofuels. 2021 Mar 23;14:76. doi: 10.1186/s13068-021-01928-8 (PMC7986309; doi:10.1186/s13068-021-01928-8)
Supplement: Supplementary file 1 — Additional file 1. Three CeDGAT gene cDNA sequences. [file 13068_2021_1928_MOESM1_ESM.docx]

Additional file1

**Three *CeDGAT* genes cDNA sequences**

**The completed ORF of *CeDGAT1* sequence**

ATGGCCAACTCTCTCGATTCCCACCATCCCCACCCCCACACCCAGACGCAAACCCAATCCACATCCCAATCCCAATCCCAATCCGACAAAGCCTCCTCTCTCCGCCACCGCCATCCTGCTCACCCCGGGGACCAGGACTCCTCCTCCCCCATGCCCCCTACCGTCTCCGATTCCGCCATCCCAGATCAACCTTCCATCGATGGCCATGGCCTACCACCACCGCCTTCCCAGGTGCCTCCGCAGTATCGCGCCTCAGCTCCAGCTCACAGGCGCATCAGAGACAGCCCCCTCAGCAATGAAGCTATTTTTAGACAGAGCCATGCGGGTCTTCTTAATCTGTGCATTGTTGTGCTTATTGCGGTCAATAGCCGCCTTATTATTGAGAATATCATGAAGTATGGGTTACTAATAAGGGCAGGATTTTGGTTTAGTGCGAGATCATTAAGGGATTGGCCTCTTCTCATGTGTTGCCTGAGTCTCCCCATTTTTCCTCTTGCCTCATTTTTGGTTGAGAAGATGGCAGACCGCAAGCGCATATCTGAGCCTATAGTTATTCTTTTGCAAATTTTTATAACAACCGCGGCCATTGTGTATCCGGTTATTGTAATTCTCAAGTGTGATTCAGCAGTCTTGTTTGGATTTGTTTTAATGTTCATTGCATGCATTCTTTGGATGAAGCTTGTATCATATGCCCATACAAACCATGATATCAGAGCTTTGTTTAAAACTATTGATAAGGATTCTCTCAGCAATAATTCATTTGATCTTGAAAACTTGAAAGGGCCTACTTTTAAAAGCCTTGTTTACTTTCTCTTAGCTCCTACTCTTTGTTATCAGCCTAGCTATCCCCGCACTGCTTCTATCAGAAAGGGTTGGGTTCTTCGTCAGCTTGTGAAGTGTATCGTTTTTACTGGTTTGATGGGATTTATAATTGAACAGTATATAAACCCGATAGTGCAGAATTCACAACACCCACTGAAAGGGAATTTTTTGAATGCCATAGAGAGGGTTTTGAAGCTATCTGTGCCTACTCTTTACGTTTGGCTATGCATGTTCTACTGCTTCTTCCATCTCTGGCTAAATATTCTAGCGGAGCTACTGCAGTTTGGAGACCGGGAATTTTATAAAGATTGGTGGAATGCAAAAACTATTGAAGAGTATTGGAGAATGTGGAACATGCCGGTGCATAAATGGATGATCCGTCATGTATATTTCCCTTGCCTAAGGCTTGGCTTATCAAAGGGTGCTGCTCTTCTAATCTCGTTTTTGATTTCTGCTTTGTTCCATGAGGTTGTAGTTGGGGTTCCTTGTCATATATTGAAGTTTTGGGCTTTCATCGGTATAATGTTCCAGATTCCTTTGGTTTTCTTGACAAAGTATCTTCAAGACGAGTTCAGAAGTTCAATGGTTGGAAACATGATATTTTGGTTCTTCTTTAGCATATTGGGGCAGCCAATGTGTGTTCTCTTATATTACCATGATGTCATGAATAGAAAAGGC AGAACATAG


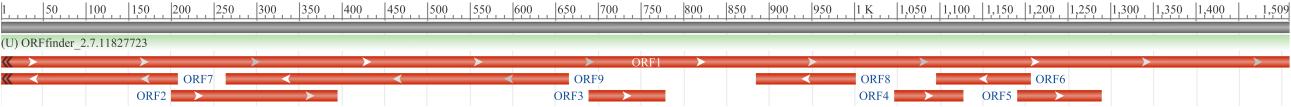


**Additional fig1 Schematic of *CeDGAT1* ORF in NCBI**

**The completed ORF of *CeDGAT2-1* sequence**

ATGACGGAGGCTAACGGTAACGGAGAGAACTTAACGGCGGCAACAACGAACGGATCTTTAACGGAGGGTGGGCCGAAGAAAGAGGCCGCCTTCACCGGCACCGAGTACTCCCCGTGGAAGACCTTCCTGGCCATCACCGTCTGGCTCGGGATGATCCACGCCAATGTCGTCCTCGTGCTGCTGGCCCTCTTTGTCCTCCCCCCGCGCATTGCCCTGATGGTCATTGCGGTGCAGCTGGTGTTCATGGTGGTACCGGTGGATCGTAAGAGTGAACTCGGTAAGAAATTGGCCAGATTTGTGTGTAGATATGCTTGTGGGTATTTCCCGATCACGCTTCATTTGGAGGATTATAATGCATTTGATCCAAAGCAAACCTACGTGTTTGGATATGAGCCACACTCAGTTTTGCCGATTGGAATTTGTATACTATCTGATAATGCGGGTTTCATGCCTCTTCCTAAAACCTTGGCCCTTGCTAGCACCGCTGTATTCTGGACACCATTTCTGAGGCAGATATGGACATGGATGGGCCTGGTTCCTGCTTCTAGACAGAGCTTTTATTCAAATCTAGCTCGTGGGAATAGTTGTGCTGTCATACCGGGTGGTGTTCAGGAGATGCAATATATGGACTCAACTTCTGAGGTCGCCTTTCTTAAAGCAAGAAAAGGATTTGTGAAAGTAGCAATCGAAATGGGCAGCCCCCTCGTGCCAACCTTTTGTTTCGGTCAGAGACATGTATACAAGTGGTGGAGGCCTGAAGGGAAAATATTTGTGAAGATCTCTAGAGCCATCAAGTTCACTCCACTTGTATTTTGGGGAAGATGGGGGACACCCATTCCATACCGAGTGCCCATGCATGTGGTGGTTGGTACTCCCATTAAAGTCAAGCAAAATCCCCAACCTACCTATGATGAGATAAACGAAGTGCATGCGCAGTTTTTGGAAGCAATGGAGAAGCTCTACGAAAAATACAAGGGCCGTTTTGGTTATGATGAGCTCCCTCTTAGAATTTTATAG


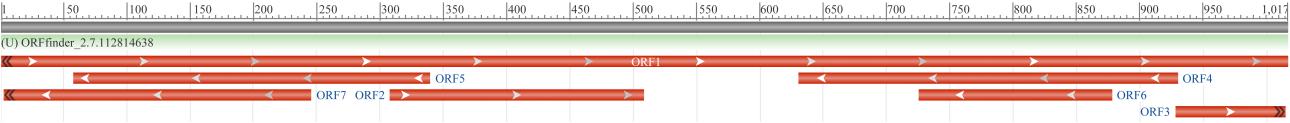


**Additional fig2 Schematic of *CeDGAT1* ORF in NCBI**

**The completed ORF of *CeDGAT2-2* sequence**

ATGGGAAACAAGGAAACAATGGAAACACCCAAAACTAATACAAGGCCACCACTGCTGAAGAGCATAGTGGCCCTCTTCCTATGGATGGGTATCATACAGCTCAATGTTGTGCTACTGGTAACGGCCCTCTTCGTCCCGCTTCGCATCGGCACCATGATTATTGGGTTTCTTGTTTTGATGAGGTTGTTGCCTGTCAATCCTAAGAGCAAATTTGGAAGCAAAGTTGCAAAGTTTATAGACAAAAATGCATATGGATATTTCCCAATTACAGTTCATTTGGAGGATGAAAAGGCTTTTGATCCCAACCAAGCTTATGTATTTGGGTATGAGCCGCACACAATATATGCTCTTGGAGCATGGGCACTAACAGACCGCAGCAGCTTGGCACCTGTACCAAAGATCAAGTTTACTGCTGCCAGCATTGCATTCAACATTCCAATTCTGAGGCATATTTGGACATGGCTGGGTCTTGTTCCCGTAACCAGAAAGAGTTTCATCAAGCAATTGTCAGCCGGAAACAGCTGCATTGTTGTACCAGGTGGTGTTCAAGAGATGCTTCATTTGGAGCAGGATTCAGAGGTTGCTTTTCTGAAATCAAGGAAGGGATTTGTAAAATTGGCAATTGAGATGGGCTCCCCACTTGTCCCTGTTTTCTCCTTTGGTCAGAGCCAAGCATACAAATGGTGGAGACCTCAAGGAAAAATATTTCACAAAATTTATAAGATAGTCAAGCAGCCAATAGTTTTATCCTGGGGAAGATTTGGGTCACCAATTCCATTTCGAGTACCAATGCACATTGTGATAGGTAAACCAATTCAGCTCAAGAAGAATGCTCAGCCTACTAATGATGAGGTGAATGAAGTGCATGCACAATTTGTAGCGGCAATGCAGGAGCTATTTGAGAAACACAAAGCTCAGTATGGTTCCAAAGATCTTCAACTGAGAGTGTTGTAG


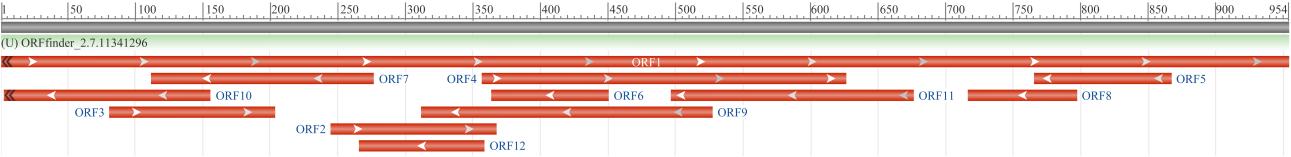


**Additional fig3 Schematic of *CeDGAT1* ORF in NCBI**
