# Supplementary material for: Ectopic overexpression of a type-II DGAT (CeDGAT2-2) derived from oil-rich tuber of Cyperus esculentus enhances accumulation of oil and oleic acid in tobacco leaves
Source: Biotechnol Biofuels. 2021 Mar 23;14:76. doi: 10.1186/s13068-021-01928-8 (PMC7986309; doi:10.1186/s13068-021-01928-8)
Supplement: Supplementary file 2 — Additional file 2. Physical and chemical properties of CeDGAT protein analysis in Cyperus esculentus L. [file 13068_2021_1928_MOESM2_ESM.docx]

Additional file2

**Additional Table1 Physical and chemical properties of CeDGAT protein analysis in *Cyperus esculentus L.***

| Protein name | AtDGAT1 | CeDGAT1 | AtDGAT2 | CeDGAT2-1 | CeDGAT2-2 |
| --- | --- | --- | --- | --- | --- |
| Length(aa) | 520 | 502 | 314 | 338 | 317 |
| Molecular weight(KD) | 58.98 | 57.68 | 35.85 | 38.08 | 35.75 |
| PI | 8.85 | 9.23 | 9.00 | 9.30 | 9.93 |
| Instability index | 45.42 | 53.33 | 43.43 | 37.52 | 39.84 |
| Aliphatic index | 99.35 | 102.93 | 97.77 | 90.83 | 102.68 |
| GRAVY | 0.242 | 0.018 | 0.190 | 0.119 | 0.130 |
| TMHMM | 9 | 8 | 2 | 1 | 1 |
| Predicted locations | Endoplasmic reticulum | Endoplasmic reticulum | Cellular ER | Cellular ER | Cellular ER |
