# Supplementary material for: Ectopic overexpression of a type-II DGAT (CeDGAT2-2) derived from oil-rich tuber of Cyperus esculentus enhances accumulation of oil and oleic acid in tobacco leaves
Source: Biotechnol Biofuels. 2021 Mar 23;14:76. doi: 10.1186/s13068-021-01928-8 (PMC7986309; doi:10.1186/s13068-021-01928-8)
Supplement: Supplementary file 3 — Additional file 3. Primers used in this study. [file 13068_2021_1928_MOESM3_ESM.docx]

Additional file3

**Additional Table2 Primers used in this study**

| Primer | Sequence（5’-3’） |
| --- | --- |
| *CeDGAT1*-F | ATTGTTGTGCTTATTGCGGTCA |
| *CeDGAT1*-R | GCGGTCTGCCATCTTCTCA |
| *CeDGAT2-1*-F | TGACGGAGGCTAACGGTAACG |
| *CeDGAT2-1*-R | AGCAGCACGAGGACGACATT |
| *CeDGAT2-2-*F | TGAGGTTGTTGCCTGTCAATCC |
| *CeDGAT2-2-*R | CTATTGATGGCGCTTGAATATG |
| *18S rRNA-*F | CTACGTCCCTGCCCTTTGTACA |
| *18S rRNA-*R | ACACTTCACCGGACCATTCAA |
| *ORF-CeDGAT2-2*-F | ATGGGAAACAAGGAAACAATGGA |
| *ORF-CeDGAT2-2*-R | CTACAACACTCTCAGTTGAAGAT |
